# The Reliability of Linear Speed with and without Ball Possession of Pubertal Soccer Players

**DOI:** 10.3390/jfmk8040147

**Published:** 2023-10-16

**Authors:** Nikolaos Manouras, Christos Batatolis, Panagiotis Ioakimidis, Konstantina Karatrantou, Vassilis Gerodimos

**Affiliations:** Department of Physical Education and Sports Science, University of Thessaly, 42100 Trikala, Greece

**Keywords:** test–retest reproducibility, evaluation, sprint, performance index, dribbling speed, team sports, developmental years

## Abstract

Reliable fitness tests with low day-to-day and trial-to-trial variation are a prerequisite for tracking a player’s performance or for identifying meaningful changes in training interventions. The present study examined the inter- and intra-session reliability of 30 m linear speed with and without ball possession as well as the reliability of a specific performance index of pubertal soccer players. A total of 40 pubertal (14.87 ± 1.23 years old) male soccer players performed two testing sessions (test–retest) separated by 72 h. Both testing sessions included a protocol consisting of two maximal trials of 30 m linear speed with and without ball possession. A performance index, indicating the difference between the two speed tests, was also calculated using two different equations (delta value and percentage value). The relative and absolute inter-session reliabilities were good/high for all testing variables (ICC = 0.957–0.995; SEM% = 0.62–8.83). There were also good/high relative and absolute intra-session reliabilities observed for all testing variables (ICC = 0.974–0.987; SEM% = 1.26–6.70%). According to the Bland–Altman plots, the differences between test–retest and trials for all observations were within the defined 95% limits of agreement. The reliable testing protocols and performance index for the evaluation of linear speed with and without ball possession, observed in this study, may be used in speed monitoring and training planning of pubertal soccer players.

## 1. Introduction

Soccer is, mainly, a sport that depends on aerobic metabolism because of its 90 min match duration, but the most crucial actions in a soccer game (i.e., sprinting, jumping, change of direction, and kicking and dribbling a ball) involve the anaerobic metabolic system [1,2,3,4,5,6]. One of the most important technical skills is sprinting while keeping control of the ball (dribbling the ball), which is considered a hallmark of gifted soccer players [7,8] and decides the outcome of the game [9,10]. According to the official data, each player covers 10,627–12,027 m in a soccer game of which 119–286 m is covered with a ball in possession, depending on the tactical position role of each player [11]. There is also evidence that elite soccer players cover 215–446 m at a top speed of >23 km/h in a soccer game [11]. Therefore, the reliable and valid evaluation of speed with and without a ball in possession in soccer players may be used for physical fitness monitoring (depicting the current physical strengths and weaknesses) and training planning of young soccer players.

Several studies in the scientific literature have examined the validity and the reliability of linear sprint test, using different distances (mainly 5–40 m), in young adults [12] as well as in pre-pubertal and pubertal soccer players [13]. Furthermore, linear sprint tests have shown moderate-to-high intra-session reliability (reliability between trials at the same testing occasion) and inter-session reliability (reliability between the first and the second testing occasions, i.e., test–retest) with ICC values that ranged from 0.57 to 0.98 in young soccer players (using different age groups from U11 to U18) [14,15,16,17,18,19,20,21,22]. Different factors such as the distance of the sprint, the measured system, the testing protocol, and the time interval between test–retest measurements may affect the reliability of sprint test [16,23,24,25,26,27,28,29]; however, future studies are needed to strengthen these findings. Other factors that could affect the reliability of measurement during the developmental years are the age and the maturation stage. Buchheit et al. [14] demonstrated that the age or the maturation stage did not seem to clearly affect the test–retest reliability of linear sprint test in young soccer players from U13 to U18 (although ICC values were different among age groups, the effect size of differences was small). In the same context, Dugdale et al. [17] examined the reliability of 10 m and 20 m linear sprint tests in different age groups of soccer players (U11–U17) and reported lower ICC values in U12 and U17 vs. other age groups; however, the effect size differences were small.

Regarding the reliability of dribbling tests, the greater proportion of studies have examined the reliability of different dribbling agility tests (i.e., Illinois dribbling test, 505 CoD test, UGhent dribbling test, zigzag dribbling test, Bangsbo and Mohr short dribble test, and slalom dribble test) in young soccer players, reporting moderate-to-high reliability [7,9,13,18,20,22,30,31,32], while few studies have assessed the reliability of the linear dribbling speed [18,22,32]. However, the linear sprint is of crucial importance in soccer because it supports the player in creating a chance to score [33]. This notion has been strengthened by a previous study which determined that scoring players (*n* = 161) performed linear sprints prior to 45% of all analyzed goals [34]. Previous study [22], which examined the intra-session reliability of linear dribbling speed during a 30 m sprint in 25 young male soccer players aged 15–18 years, reported a high reliability among trials (ICC = 0.88). Similarly, another study [18] that examined the reliability of 20 m linear dribbling speed in young soccer players (10–12 years old) also found a high reliability among trials (Cronbach a = 0.85).

Except for the measurement and evaluation of linear speed with ball possession, which is affected by the level of the player’s dribbling technique, a reliable calculation of a specific performance index, which demonstrates the difference between linear speed with and without ball possession, is important especially after the age of 13–14 years, as at that instance the technique is automated. Moreover, the reliable calculation of the difference between the two tests (linear speed with and without ball possession) may be used to provide important information about how much the ball handling technique reduces the soccer player’s time required for a sprint, helping soccer coaches to plan training appropriately to eliminate this difference. To the best of our knowledge, no previous study has examined the intra-session and inter-session reliability of such an indicator in pubertal soccer players.

Considering all the above facts, the main objectives of this study were as follows:

(a) To examine the inter-session reliability (reliability between the first and the second testing occasions, i.e., test–retest) as well as the intra-session reliability (reliability between trials at the same testing occasion) of 30 m linear speed with and without ball possession;
(b) To calculate (using two different equations: delta value and percentage value) and examine the intra-session reliability and inter-session reliability of a specific performance index that indicates the difference between the two speed tests (linear speed with and without ball possession) in pubertal soccer players.

## 2. Materials and Methods

### 2.1. Participants

Forty pubertal male soccer players (age: 14.87 ± 1.23 years; tanner stage: 3.3 ± 0.46; body height: 169.19 ± 0.38 cm; body mass: 60.61 ± 11.42 kg) who were members of different soccer academies and played at different positions (14 defenders, 16 midfielders, and 10 forwards) volunteered to participate in the current study. All the participants were soccer players from different soccer academies of the region of Thessaly and participated in national soccer championships in the under 16 category. It should be mentioned that the sample of the study was selected through the “Union of Trikala Soccer Academies” from different soccer academies where the soccer players met the appropriate inclusion criteria. The inclusion criteria were that participants should be males, of pubertal age (13.5–16 years old), i.e., in the under 16 category, and healthy with no injury in the upper and lower limbs for at least 6 months before the commencement of the study. The participants should also have more than five years of experience in playing soccer, and they should be training three times per week and should have played at least one official match. Before the testing, the participants and their parents were informed about the evaluation procedure, and they provided their written consent. The study was conducted according to the Declaration of Helsinki and approved by the Ethics Committee of the University of Thessaly.

### 2.2. Measures

All speed-testing procedures were performed on a natural soccer turf, and the participants wore their soccer footwear during the test. During both testing occasions (test and retest), the participants performed a standardized 25 min warm-up. The first 15 min of the warm-up included 8 min of running exercises (running straight ahead, running with hip out and hip in, running with circling partner, and running with shoulder contact) and 7 min of neuromuscular exercises and sub-maximal speed trials (i.e., skipping, butt kicks, carioca drill, forward and backward running, and submaximal sprints of 20 m). The other 10 min included technical exercises with a ball (running with a ball, team passing, pass and move, and dribble–pass–move) and dynamic stretching (straight leg march, high knees, lunges with torso twists, front swings, side cross swings, and hip in, hip out, and lateral lunges).

Afterward, the participants’ 30 m linear speed with and without ball possession was evaluated using a photocell timing system (Newtest 300 series Powertimer, 2000, Oulu, Finland) [35]. The photocell timing system (Newtest) that we used in the present study is widely used in scientific literature to evaluate the speed ability in different populations [36]. According to the manufacturer, the Newtest Powertimer photocell system exhibits a 0.001 s error over a 5 m sprint at a speed of 10.0 m/s [37]. Furthermore, previous studies showed that the Newtest Powertimer photocell system is a reliable instrument for speed measurement [36,37], since it did not show any marked systematic bias, and the random error associated with it was negligible [37].

During the linear speed test without a ball, the participants started from a standing position of 0.3 m behind the starting line. Photocells were positioned at 0 and 30 m along the soccer field. The testing protocol consisted of two maximal trials with a rest period of 3 min between trials. The best recorded sprint time (in seconds) was used for analysis. During the linear speed test with a ball, the ball was placed 0.3 m behind the starting line, and photocells were positioned at 0 and 30 m along the field. The participants were instructed to maintain contact with the ball in every step or every two steps; otherwise, the trial was considered invalid. The testing protocol consisted of two maximal trials with a rest period of 3 min between trials. The best recorded sprint time (in seconds) was used for analysis.

For both testing occasions (test and retest), the performance index was calculated as the difference between the two tests (linear speed without a ball and linear speed with a ball) using two different equations: (a) delta score = (best sprint time with a ball − best sprint time without a ball) and (b) % difference = [(best sprint time with a ball − best sprint time without a ball) ÷ best sprint time without a ball] × 100.

### 2.3. Design and Procedures

The testing procedures were performed at the start of the competitive season and lasted three days for each participant. During the first day, the participants were familiarized with the testing procedures. Moreover, assessments regarding biological age and anthropometric characteristics (body height and body mass) and the completion of a medical history form were completed on the first day. The assessment of biological age was performed with self-estimation using Tanner’s sexual maturation stages and was determined according to pubic hair development [38]. The body mass was also measured to the nearest 0.1 kg using a calibrated physician’s scale (Seca model 755; Seca, Hamburg, Germany), while the body height was determined to the nearest 0.1 cm using a telescopic height rod (Seca model 220; Seca, Hamburg, Germany). During the second (test) and third (retest) days, participants’ 30 m linear speed with and without ball possession was assessed, with 5 min rest interval between the two tests. The order of testing linear speed with and without ball possession was randomized; however, each participant performed the tests in the same order at both test and retest testing occasions. A computer-generated list of random numbers was used for the allocation of sequence during testing occasions. Both tests (test and retest) were performed in a soccer field, by the same investigator who was also an experienced soccer coach in the developmental ages, at the same time of the day (3–5 p.m.) and under similar environmental conditions (26–28 °C), while the duration between the two tests was 72 h. Participants were asked to follow their normal diet for two days before the study, abstain from intense exercise activity for 48 h before the study, and to have sufficient rest the night before the study.

### 2.4. Statistical Analysis

All data are presented as mean ± SD and were analyzed using IBM SPSS Statistics v.26 software (IBM Corporation, Armonk, NY, USA). A statistical power analysis (software package GPower 3.0) before the initiation of the study indicated that a total number of 30 participants would yield adequate power (>0.85) and level of significance (<0.05). In the present study, the final sample comprised 40 pubertal soccer players. The normality of data was examined using the Shapiro–Wilk test (all the data followed normal distribution). The inter-session reliability was used to evaluate the reliability between the first and the second testing occasions (test–retest reliability) using the best of the two testing trials, while intra-session reliability was used to examine the reliability between trials on the same testing occasion.

The inter-session and intra-session reliabilities were examined using indicators of both relative (intraclass correlation coefficient—ICC) and absolute reliabilities (standard error of measurement in absolute terms, i.e., SEM, and relative terms, i.e., SEM% and 95% limits of agreement - 95% LOA). We calculated ICC for single measures using a two-way random effect model for absolute agreement for the computation of ICC. The ICC value varies between 0 (indicating no reliability) and 1 (indicating perfect reliability). An ICC value of (a) below 0.5 indicates poor reliability, (b) between 0.5 and 0.75 indicates moderate reliability, (c) between 0.75 and 0.90 indicates good reliability, and (d) above 0.9 indicates high reliability [39]. The SEM quantifies the precision of individual scores on a test and is expressed in the actual units of the original measurement. Furthermore, the SEM may be presented as a percentage value (SEM%) by dividing the mean of the two measurements (test and retest) and multiplying by 100 [40]. A SEM value of (a) below 5% indicates high reliability, (b) above 5% and below 10% generally denotes good reliability, (c) equal to 10% indicates moderate reliability, and (d) above 10% indicates low reliability. The 95% LOA represents the 95% likely range for the difference between the subject’s scores in two tests [41]. The range defined by the LOA (upper and lower limits) is regarded as a reference range for changes between pairs of measurements (when the differences between values range within the defined limits of agreement, this generally denotes a good agreement) [41]. The intertrial agreement was also examined graphically by plotting the difference between test and retest as well as the difference between trials on the same testing occasion against their mean, according to the Bland and Altman approach [42]. The Bland–Altman graph expresses good agreement between two measurements, when the differences between measurements for all observations lie within 1.96 SD [42]. The Bland–Altman plots show the measurement error schematically and help to identify the presence of heteroscedasticity (a positive relationship between the degree of measurement error and the magnitude of the measured value). Heteroscedasticity was also tested using a Pearson correlation test to examine whether the absolute intertrial difference (systematic bias) is associated with the magnitude of the measurement. The systematic bias was also calculated as the intertrial difference between test and retest values as well as the difference between trials on the same testing occasion. Finally, paired *t*-tests were also used to determine possible significant differences in linear speed with and without ball possession between test and retest as well as between trials on the same testing occasion. The level of significance was set at *p* < 0.05.

## 3. Results

### 3.1. Inter-Session Reliability 

Test and retest values (mean ± SD), as well as relative and absolute reliability indices (ICC, SEM, SEM%, 95% LOA), are presented in Table 1. Non-significant differences between test and retest values were observed (*p* > 0.05). The relative reliability between the test and retest values was high for all testing variables (linear speed with and without ball possession and performance index) with ICC values that ranged from 0.957 to 0.995.

The SEM% values denoted a high reliability for linear speed without ball (SEM% = 0.62) and linear speed with ball (SEM% = 1.69), and the performance index demonstrated higher SEM% values (8.10% expressed as a delta score and 8.83% expressed as a percentage), thereby reporting a good reliability.

**Figure 1:**
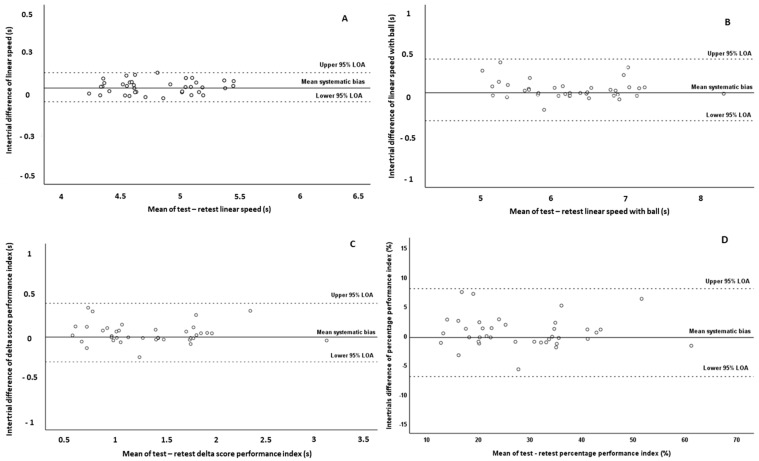
Bland–Altman plots of the linear speed test without ball possession (**A**), linear speed test with ball possession (dribbling) (**B**), performance index expressed as a delta score (**C**), and performance index expressed as a percentage value (**D**) in test and retest measurements. The central solid line characterizes the mean difference between test and retest values (systematic bias), while the upper and lower dashed lines characterize the upper and lower 95% limits of agreement, LOA (intertrial mean difference of ±1.96 SD of the intertrial difference), respectively.

Furthermore, the systematic bias was 0.04 s for linear speed with and without ball possession, −0.004 s for the performance index expressed as a delta score, and −0.34% for the performance index expressed as a percentage value. However, it should be mentioned that no presence of heteroscedasticity was observed since the absolute intertrial difference (systematic bias) was not associated with the magnitude of the measurement according to Pearson correlation test (*p* = 0.380–0.989). Thus, all variables were found to be homoscedastic. The Bland–Altman plots graphically present the reliability patterns for the assessment of linear speed with and without ball possession as well as of performance index (Figure 1). According to Bland–Altman plots, the differences between test–retest values for all observations were within the defined 95% LOA in all tested variables. However, it should be noted that the observations in linear speed without ball demonstrated the least dispersion in the Bland–Altman plot (Figure 1A), while the performance index expressed as a percentage value demonstrated the greatest dispersion (Figure 1D).

### 3.2. Intra-Session Reliability 

**Figure 2:**
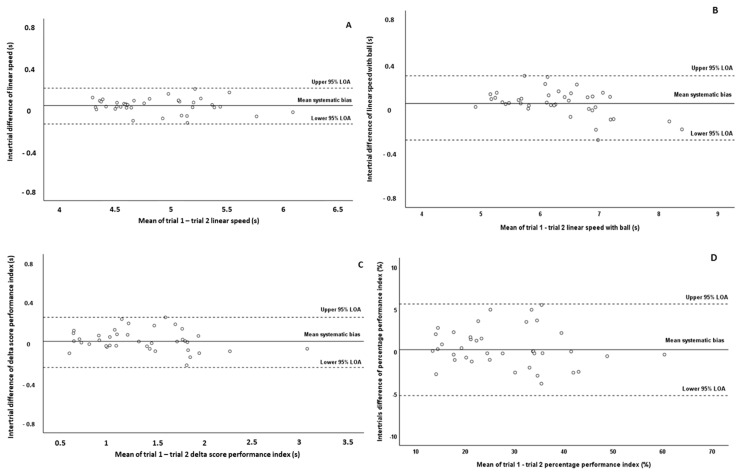
Bland–Altman plots of the linear speed test without ball possession (**A**), linear speed test with ball possession (dribbling) (**B**), performance index expressed as a delta score (**C**), and performance index expressed as a percentage value (**D**) in test and retest measurements. The central solid line characterizes the mean difference between test and retest values (systematic bias), while the upper and lower dashed lines characterize the upper and lower 95% limits of agreement, LOA (intertrial mean difference of ±1.96 SD of the intertrial difference), respectively.

Test and retest values (mean ± SD), as well as relative and absolute reliability indices (ICC, SEM, SEM%), are presented in Table 2. Non-significant differences between trials for all testing variables were observed (*p* > 0.05). The relative reliability among trials, according to ICC values (ICC = 0.974–0.987), was high for all testing variables. The SEM% values were also denoted a high reliability for linear speed without ball (SEM% = 1.26) and linear speed with ball (SEM% = 1.45), while the performance index demonstrated higher SEM% values (6.17% expressed as a delta score and 6.70% expressed as a percentage), thereby reporting good reliability. Furthermore, the systematic bias was 0.04 s for linear speed without ball, 0.05 s for linear speed with ball possession, 0.02 s for the performance index expressed as a delta score, and 0.21% for the performance index expressed as a percentage value. However, it should be mentioned that no presence of heteroscedasticity was observed since the absolute intertrial difference (systematic bias) was not associated with the magnitude of the measurement according to Pearson correlation test (*p* = 0.28–0.40). Thus, all variables were found to be homoscedastic. The Bland–Altman plots graphically present the intra-session reliability patterns for the assessment of linear speed with and without ball possession as well as of performance index (Figure 2). According to Bland–Altman plots, the differences between trials for all observations were within the defined 95% LOA in all tested variables. However, it should be noted that the observations in linear speed without ball demonstrated the least dispersion in the Bland–Altman plot (Figure 2A), while the performance index expressed as a percentage value demonstrated the greatest dispersion (Figure 2D).

## 4. Discussion

This study examined the inter-session reliability (reliability between the first and the second testing occasions, i.e., test–retest) as well as the intra-session reliability (reliability between trials on the same testing occasion) of linear speed with and without ball possession using different relative and absolute reliability indices (ICC-95% CI, SEM, SEM%, 95% LOA). However, the most important aspect of this study is that it calculated and tested the reliability of a specific performance index, indicating the difference between the two tests (linear speed without and linear speed with ball possession). The main finding is that there are high inter-session and intra-session reliabilities obtained for both linear speed with and without ball possession. Additionally, there are also good/high inter-session and intra-session reliabilities obtained for both performance index methods (delta score and percentage value) used for the calculation of the difference between the two tests. However, it should be mentioned that the relative reliability of the calculated performance index is lower (with greater SEM% values, although acceptably reliable) compared to that observed for absolute scores of linear speed with and without ball possession.

Our results demonstrated a high reliability of the 30 m linear speed test without ball in pubertal soccer players, where the ICC values were 0.98 and 0.995 and the SEM% values were 1.26 and 0.62 for the intra-session reliability and the inter-session reliability, respectively. Previous studies that examined the reliability of linear speed test in young soccer players reported moderate-to-high reliability with a wide range of ICC values that ranged from 0.57 to 0.98 in young soccer players [14,15,16,17,18,19,20,21,22]. There is evidence that the distance of sprint test may affect the intra-session and inter-session reliabilities (higher reliability with increasing sprinting distance), although further studies are needed to draw more reliable conclusions on this topic. For example, in a previous study, ICC values of 0.87 and 0.97 have been reported for 5 and 20 m, respectively [16]. The testing protocol used could also be an additional factor that could affect the reliability of linear speed. Previous studies that demonstrated a lower reliability of linear speed integrated linear speed testing into complex tests [23] or match simulation protocols [26,28] or required the players to adopt a defined running velocity at the start line [27]. Additionally, previous studies, demonstrating a lower reliability, inferred that the time interval between test–retest measurements (the reliability decreases with the increasing time interval between measurements) [24] as well as the measured system (more consistent results were obtained for timing lights and radar guns compared to global positioning systems where the results vary [25,29]) could affect the reliability of linear speed. In our study, we chose the Newtest Powertimer photocell system that is a reliable instrument for speed measurement [36,37] as well as a small time interval between test and retest measurements (72 h) to strengthen the reliability of the measurement.

Several studies, in the scientific literature, have used different agility tests to evaluate dribbling performance in young soccer players and have reported moderate-to-high reliability [7,9,13,18,20,22,30,31,32]. On the other hand, limited studies have focused on the reliability of linear speed with ball possession, reporting good-to-high reliability (ICC–r = 0.85–0.98 [18,22,32]; this finding is in agreement with the results of the present study, where the ICC (0.987 for intra-session and 0.982 for intersession) and SEM% (1.45 for intra-session and 1.69 for intersession) values were high. Additionally, in the present study, we observed high ICC values for both intra-session reliability (ICC = 0.974–0.979) and inter-session reliability (ICC = 0.957–0.965) of the performance index that we calculated to indicate the difference between the two tests (linear speed and dribbling speed). However, the SEM% values that we observed for the performance index were higher (SEM% = 6.17–8.83; reporting good reliability) compared to those observed for the absolute values of linear speed with and without ball possession (SEM% = 0.62–1.69; reporting high reliability). Furthermore, the performance index (especially which is expressed as a percentage value) demonstrated the greatest dispersion in the Bland–Altman plots compared to the absolute values of linear speed with and without ball possession. The lower absolute reliability (greater SEM% values) and the greater dispersion in the Bland–Altman plots for the assessment of this parameter may be attributed to the fact that the performance index is a composite of two absolute scores (linear speed with and without ball possession), each possibly varying in the same or a different direction with reassessment, resulting maybe in error propagation. It should be also mentioned that both methods used for the calculation of specific performance index were almost equally reliable (similar ICC and SEM% values), although the performance index expressed as a percentage value showed greater dispersion in the Bland–Altman plots compared to the performance index expressed as a delta value. In the scientific literature, there are no similar references that assessed the reliability of this performance index in young soccer players to compare our results. Nevertheless, the results of this study are in line with previous studies that calculated and examined the reliability of other speed performance indices such as change of direction deficit (CoDD), reporting lower reliability compared to absolute scores of different agility and linear sprint tests [43,44]. However, it should be mentioned that the performance index of our study in more reliable than the CoDD index of previous studies in young soccer players. Thus, the results of this study and previous studies demonstrate that the calculated speed performance indices should be interpreted and used with more caution.

This study has some limitations that could affect its outcomes, and, as a result, their generalization. Firstly, the results of this study are clearly limited to pubertal male soccer players (13.5–16 years old) with previous experience of playing soccer of least five years. Whether these results can be generalized to other age groups (i.e., younger or older age groups), sex (females where the scientific literature is limited), or training status individuals (i.e., soccer players with less training experience in soccer) is unknown and could be examined in future studies. Moreover, the results of the present study are limited to the testing protocol (30 m linear speed with and without ball possession) as well as to the measured system (photocell timing system) used. We did not measure intermediate distances (i.e., 5, 10, 15, and 20 m) or other speed/agility test categories (i.e., change in direction and repeated sprint) with and without ball possession in order to examine their intra-session and inter-session reliabilities. Future studies could also examine and compare the reliability of these measurements using different measured systems (i.e., photocell timing system vs. radar guns vs. global positioning systems). The sample size (although it yielded adequate power as mentioned in the methods section) may be an additional limitation of this study. A larger sample could further strengthen the results of the present study. Finally, the main objective of this study was to examine the reliability of a new variable (performance index) in pubertal soccer players; however, this study did not assess other metrics, such as sensitivity, homogeneity, validity, etc. Future studies could examine, apart from reliability, and the other metrics associated with this variable (performance index) in different groups of soccer players.

## 5. Conclusions

In conclusion, both 30 m linear speed tests with and without ball possession showed high intra-session and inter-session reliabilities in pubertal male soccer players. Furthermore, the performance index (difference between the two tests), which we calculated using two different equations (delta score and percentage value), is also reliable for the evaluation of pubertal male soccer players. Reliable testing protocols for the evaluation of linear speed with and without ball possession may be used, by coaches and physical conditioning trainers, in speed monitoring and training planning of pubertal soccer players. The performance index, which we calculated in the present study (using two different equations), may be used to provide significant information about how much the ball handling technique reduces the soccer player’s time required for a sprint. Therefore, in this way, soccer coaches can design, implement, and guide appropriate training programs to eliminate this difference (sprint time of linear speed test with and without ball possession), aiming to improve dribbling technique using specialized soccer exercises.

## Figures and Tables

**Table 1:** Inter-session (test–retest) reliability indices of linear speed with and without ball possession in pubertal soccer players.

Variables	Test	Retest	ICC (95% CI)	SEM	SEM%	95% LOA	
						Lower	Upper
Linear speed without ball	4.85 ± 0.43 s	4.89 ± 0.42 s	0.995 (0.960–0.998)	0.03 s	0.62	−0.045	0.13
Linear speed with ball	6.24 ± 0.79 s	6.28 ± 0.78 s	0.982 (0.965–0.990)	0.11 s	1.69	−0.37	0.45
Performance Index							
Delta score	1.39 ± 0.60 s	1.39 ± 0.59 s	0.965 (0.934–0.981)	0.11 s	8.10	−0.43	0.43
Percent value	28.67 ± 12.22%	28.33 ± 12.05%	0.957 (0.919–0.977)	2.52%	8.83	−7	8.45

ICC: intraclass correlation coefficient, 95% CI: 95% confidence interval, 95% LOA: 95% limits of agreement, SEM: standard error of measurement, SEM%: standard error of measurement expressed as a percentage value.

**Table 2:** Intra-session reliability indices of linear speed linear speed with and without ball possession in pubertal soccer players.

Variables	Trial 1	Trial 2	ICC (95% CI)	SEM	SEM%	95% LOA	
						Lower	Upper
Linear speed without ball	4.87 ± 0.44 s	4.90 ± 0.43 s	0.98 (0.957–0.990)	0.06 s	1.26	−0.14	0.20
Linear speed with ball	6.26 ± 0.83 s	6.32 ± 0.77 s	0.987 (0.970–0.993)	0.09 s	1.45	−0.28	0.29
Performance Index							
Delta score	1.40 ± 0.63 s	1.42 ± 0.57 s	0.979 (0.961–0.989)	0.09 s	6.17	−0.24	0.25
Percent value	28.74 ± 12.47%	28.95 ± 11.48%	0.974 (0.951–0.986)	1.93%	6.70	−5.22	5.64

ICC: intraclass correlation coefficient, 95% CI: 95% confidence interval, 95% LOA: 95% limits of agreement, SEM: standard error of measurement, SEM%: standard error of measurement expressed as a percentage value.

## Data Availability

Data are unavailable due to privacy or ethical restrictions.
